# Ovarian cancer staging and follow-up: updated guidelines from the European Society of Urogenital Radiology female pelvic imaging working group

**DOI:** 10.1007/s00330-024-11300-7

**Published:** 2025-01-11

**Authors:** Stefania Rizzo, Giacomo Avesani, Camilla Panico, Lucia Manganaro, Benedetta Gui, Yulia Lakhman, Pamela Causa Andrieu, Nishat Bharwani, Andrea Rockall, Isabelle Thomassin-Naggara, Teresa Margarida Cunha, Evis Sala, Rosemarie Forstner, Stephanie Nougaret

**Affiliations:** 1 Imaging Institute of Southern Switzerland (IIMSI), Ente Ospedaliero Cantonale (EOC), via Tesserete 46, 6900 Lugano, Switzerland; 2 Faculty of Biomedical Sciences, Università della Svizzera italiana (USI), via G. Buffi 13, 6900 Lugano, Switzerland; 3 Department of Imaging and Radiation Oncology, Fondazione Policlinico Universitario A. Gemelli IRCCS, Rome, Italy; 4 Department of Radiological, Oncological and Pathological Sciences, Sapienza University of Rome, Policlinico Umberto I, Viale del Policlinico 155, 00161 Rome, Italy; 5 Department of Radiology, Memorial Sloan Kettering Cancer Center, New York, NY USA; 6 Department of Radiology, Mayo Clinic, Rochester, MN USA; 7 Department of Radiology, Imperial College Healthcare NHS Trust, London, UK; 8 Department of Surgery and Cancer, Faculty of Medicine, Imperial College London, London, UK; 9 Radiology Imaging and Interventional Radiology Specialized Department (IRIS), Tenon Hospital, Public Hospital of Paris, Paris, France; 10 Department of Radiology, Instituto Português de Oncologia de Lisboa Francisco Gentil, Lisbon, Portugal; 11 Department of Radiology, University Hospital of Salzburg, PMU, Salzburg, Austria; 12 Department of Radiology, Montpellier Research Center Institute, PINKCC Laboratory, Montpellier, France

**Keywords:** Ovarian cancer, Tomography (x-ray computed), Magnetic resonance imaging, Photon emission computed tomography, Structured report

## Abstract

**Objective:**

To provide up-to-date European Society of Urogenital Radiology (ESUR) guidelines for staging and follow-up of patients with ovarian cancer (OC).

**Methods:**

Twenty-one experts, members of the female pelvis imaging ESUR subcommittee from 19 institutions, replied to 2 rounds of questionnaires regarding imaging techniques and structured reporting used for pre-treatment evaluation of OC patients. The results of the survey were presented to the other authors during the group’s annual meeting. The lexicon was aligned with the Society of American Radiology (SAR)-ESUR lexicon; a first draft was circulated, and then comments and suggestions from the other authors were incorporated.

**Results:**

Evaluation of disease extent at diagnosis should be performed by chest, abdominal, and pelvic CT. The radiological report should map the disease with specific mention of sites that may preclude optimal cytoreductive surgery. For suspected recurrence, CT and [^18^F]FDG PET-CT are both valid options. MRI can be considered in experienced centres, as an alternative to CT, considering the high costs and the need for higher expertise in reporting.

**Conclusions:**

CT is the imaging modality of choice for preoperative evaluation and follow-up in OC patients. A structured radiological report, including specific mention of sites that may preclude optimal debulking, is of value for patient management.

**Key Points:**

***Question*** *Guidelines were last published for ovarian cancer (OC) imaging in 2010; here, guidance on imaging techniques and reporting, incorporating advances in the field, are provided.*

***Findings*** *Structured reports should map out sites of disease, highlighting sites that limit cytoreduction. For suspected recurrence, CT and 18FDG PET-CT are options, and MRI can be considered.*

***Clinical relevance*** *Imaging evaluation of OC patients at initial diagnosis (mainly based on CT), using a structured report that considers surgical needs is valuable in treatment selection and planning.*

## Introduction

Ovarian cancer (OC) is the leading cause of death from gynaecologic malignancies, with an observed number of deaths in the EU in 2017 of 26.221 and a prediction of 26.500 deaths in 2022 [1].

The ovarian, fallopian tube, and primary peritoneal cancer are considered a single entity (referred to as OC for simplicity) because of the similar staging, management, and prognosis. The International Federation of Gynaecology and Obstetrics (FIGO) system, last revised in 2014, is mostly used for staging these cancers and relies on surgical exploration to determine the stage [2]. High-grade serous carcinoma is the most common histologic subtype of OC, and most patients present with advanced-stage disease at diagnosis [3].

Currently, the mainstay of OC treatment is based on upfront surgery (complete cytoreduction), and a platinum-based combination chemotherapy [3]. The aim of surgery is complete (or at least optimal) cytoreduction. A thorough staging surgery allows for diagnosis, staging, treatment, and assessment of the overall prognosis [4]. The results of the prospective multi-centre randomised Trial of Radical Upfront Surgical Therapy in advanced ovarian cancer (TRUST) are anticipated by the end of 2024. This study is expected to clarify the optimal timing of cytoreductive surgery by comparing outcomes of primary cytoreductive surgery followed by adjuvant chemotherapy versus neo-adjuvant chemotherapy followed by interval cytoreductive surgery [5]. In the meantime, optimal primary treatment selection is best achieved through multidisciplinary collaboration between gynaecologic surgical oncologists, medical oncologists, pathologists, and radiologists.

Pre-operative imaging delineates disease extent and facilitates multidisciplinary treatment planning. This includes assessing the feasibility of upfront surgery, determining the need for intra-operative support from other surgical specialties, estimating operative time, and guiding biopsies if upfront cytoreduction is not feasible. According to the ESGO-ESMO-ESP recommendations, pre-operative imaging with contrast-enhanced CT, MRI, and [^18^F]FDG PET-CT, with a structured radiological report, may be considered for the initial evaluation of patients with advanced ovarian carcinoma, with a level of evidence IIIA [3].

The European Society of Urogenital Radiology (ESUR) published its guidelines for OC imaging in 2010 [6]. The purpose of this update is to provide radiologists with comprehensive guidance on imaging techniques and reporting for OC patients prior to treatment and during follow-up, incorporating recent advances in the field. These guidelines do not cover the assessment and characterisation of ovarian masses, which are addressed by other ESUR guidelines and by the Ovarian-Adnexal Reporting And Data System (O-RADS) US and O-RADS MRI risk stratification systems [7–9].

## Methods

### Literature search and questionnaire

The updating process has been implemented with the following steps:

Step 1: After reviewing the literature, three authors (S.R., S.N., R.F.) designed a questionnaire regarding imaging techniques used for the pre-treatment evaluation of OC.

Step 2: A structured questionnaire of 87 questions was designed, and the questions focused on imaging techniques (CT, MRI, [^18^F]FDG PET-CT) used for staging and follow-up in OC patients.

Step 3: The questionnaire was administered via an electronic link to all ESUR Female Pelvic Imaging working group members in November 2023.

Step 4: All responses were collected on a dedicated survey platform, analysed by four authors (G.A., S.R., S.N., R.F.), and presented during the ESUR Female Pelvic Imaging working group at the annual European Congress of Radiology Meeting in March 2024.

Step 5: To address contradictory responses provided by the participants, a secondary questionnaire was sent either to reach a consensus or for questions raised as open questions in the first round.

Step 6: All the answers were lastly analysed by five authors (G.A., S.R., C.P., S.N., R.F.). Each item was categorised as recommended if there was > 80% agreement among experts or optional if such agreement was not reached.

### Structured report

The proposal of a structured report was adapted according to the survey results. The survey included the ESUR lexicon [6], and during the elaboration of the paper, it was aligned with the SAR-ESUR lexicon [10].

### Paper preparation

The main authors prepared a first draft of the guidelines, incorporating all the relevant information raised by the questionnaires, during the ECR annual and online meetings. They then circulated the manuscript among all authors for comments and suggestions.

## Results

The panel (i.e., responders to both surveys) comprised 21 experts in the field of gynaecological oncology imaging from 19 institutions, including 15 European Centres (Austria (*n*  =  2), France (*n*  =  3), Germany (*n*  =  1), Italy (*n*  =  2), Portugal (*n*  =  2), Spain (*n*  =  1), Switzerland (*n*  =  2), and UK (*n*  =  2) and 4 institutions situated outside Europe, Japan (*n*  =  1) Uruguay (*n* = 1) and USA (*n*  =  2). The years of training ranged from 5–20 years of experience after fellowship.

### Recommendations

The recommendations cover indications and techniques to use for pre-treatment evaluation and follow-up, technical parameters, standardised reports, and future perspectives. The main results for recommendations are summarised in Table 1.

**Table 1:** Recommendations (Agreement > 80%) for evaluation at diagnosis and during follow-up of OC patients

**Evaluation at diagnosis**	
**Imaging technique**	
CT	**Indication**
	To confirm the presence of peritoneal disease or other metastatic sites of the disease
	To define disease extent for optimal treatment planning
	To guide percutaneous biopsy to confirm diagnosis
	**Protocol**
	Portal venous phase^a^
	No specific preparation (no fasting, no oral contrast, no attention to menstrual cycle)^a^
	MultiSlice (at least 16)
	3 mm multiplanar reconstructions
	Thorax^a^, abdomen and pelvic
MRI of the Abdomen and pelvis	**Indication**
	When CT with contrast medium is not feasible
	Further assessment of potential surgically critical disease sites
	Pregnant patients
	Evaluate the extent of serosal and mesenteric involvement
	**Protocol**
	Abdomen and pelvis
	Spasmolytic agent^a^
	Contrast medium^a^ (optional pineapple juice per os)
	T2w, T1w, DWI^a^, contrast-enhanced T1w
[^18^F]FDG PET-CT	**Indication**
	Suspected stage IVB Equivocal lymph nodes
	**Protocol**
	18F Fluorodeoxyglucose-low-dose CT (or contrast-enhanced CT if not performed as a separate exam)
**Evaluation during Follow-up**	
**Imaging technique**	
CT	**Indication**
	After the end of adjuvant treatment and during maintenance therapy
	Before interval debulking surgery (IDS)
	**Protocol**
	Similar to staging
[^18^F]FDG PET-CT	**Indication**
	If there is high suspicion but no disease on CT When secondary cytoreduction is considered^a^
	**Protocol**
	[^18^F]FDG PET-CT (or contrast-enhanced CT if not performed as a separate exam)

^a^ new compared to prior guidelines [6]

Details on CT and MRI protocols are included in the supplementary materials (Supplementary Table 1).

### Structured radiological reporting

Over 80% consensus was reached for organ-based structured reporting. The lexicon was aligned to the SAR-ESUR lexicon for consistency, as shown in Table 2. A table highlighting the differences between the 2010 and 2024 guidelines is included in the supplementary materials (Supplementary Table 2). For lymph node metastases definition, the 2020 guidelines [6] and the node-RADS were referred to [11]. Internal mammary lymph nodes that were not mentioned in the node-RADS were included with the cardio-phrenic and retrocrural lymph nodes for size reference.

**Table 2:** Radiological report according to anatomical sites (chest, abdomen, and pelvis), with description and explanation of site importance

Anatomical sites	Description	Why
**Chest**		
Pulmonary nodules	– Metastases – Non-oncological findings: infectious/inflammatory abnormalities, interstitial/fibrotic changes	– FIGO STAGE IVB, thoracic surgeon evaluation – Pulmonary or infectious disease specialist evaluation
Thoracic lymphadenopathy (follow the Node-RADS criteria [11])	– Supraclavicular, mediastinal, hilar, axillary (short-axis diameter ≥ 10 mm) Internal mammary, cardio-phrenic, retrocrural lymph nodes (short axis ≥ 5 mm	– FIGO STAGE IVB, may need a thoracic surgeon evaluation
Pleural metastases	– Yes/no	– FIGO STAGE IVB, thoracic surgeon evaluation
Pleural effusion	– Yes/No (if yes, small/medium/large volume, subjectively assessed [10])	– FIGO STAGE IVA Drainage can be neededCytology may be warranted
Other thoracic findings	– Non-oncological findings: cardio-vascular abnormalities, other anatomical variants	
**Abdomen and pelvis**		
Liver	– Perihepatic implants – Subdiaphragmatic implants – Hepatic parenchyma: metastasis – Porta hepatis	– FIGO STAGE III C, potentially resectable disease; describe if deep extension into the parechyme, because hepato-biliary surgeon evaluation for hepatic wedge resection can be required – FIGO STAGE IVB; if metastasis(es) are central or multisegmental in the parenchyma, they are considered **difficult to resect** – Can be difficult to resect
Spleen	– Perisplenic implants – Splenic parenchyma metastasis	– FIGO STAGE III C, usually resectable disease, but better to plan splenectomy in advance to administer immunisations
Pancreas	– Infiltration of any part of the pancreas	– May preclude optimal cytoreduction – Maybe resected together with the spleen
Diaphragm	– Right/Left/Bilateral	– May need thoracic surgical evaluation. Diaphragmatic surface along the bare area of the liver may be difficult to visualise, and the surgeon may need more time to explore these regions to achieve complete cytoreduction
Peritoneal carcinomatosis outside the pelvis	– paracolic gutters, gastro-colic ligament, gastro-splenic ligament, lesser omentum, lesser sac	– May preclude optimal cytoreduction, according to the gynaecologist experience, and hepato-biliary surgeon evaluation may be required
Mesentery	– Root of mesentery involvement – Pattern: nodular (mention lesion size) or infiltrative (retractile)	– Usually preclude optimal cytoreduction – May preclude optimal cytoreduction
Greater omentum	– Thickness measurement – pattern (reticulonodular, nodular, or omental cake) – Possible bowel infiltration	– Helpful for the surgical planning
Peritoneal carcinomatosis in the pelvis	– Paravesical spaces, rectum, pelvic sidewall, pouch of Douglas	– Helpful for the surgical planning
Abdominal wall	– Depth of the extension	– May preclude optimal cytoreduction
Bowel/stomach invasion	– Involvement and number of the implants	– May preclude optimal cytoreduction**;** diffuse carcinomatosis of the bowel involving more than 3 colon segments or such large small bowel parts that resection would lead to a short bowel syndrome (remaining bowel < 1.5 m),
Lymphadenopathy (follow the Node-RADS criteria [11])	Abdominal lymph nodes (short axis ≥ 10 mm) – Retroperitoneal above the renal arteries (supra-renal para-aortic, inter-cavo-aortic) – Retroperitoneal below renal arteries (infra-renal para-aortic, inter-cavo-aortic) – Mesenteric – Pelvic (common iliac, external iliac, internal iliac, obturatory, parametrial, mesorectal) Inguinal lymph nodes (short axis ≥ 15 mm)	– Upper abdominal lymph nodes may preclude optimal cytoreduction, if the vessels are infiltrated; vascular surgeon evaluation can be requested – FIGO STAGE IVB, but they may be removed surgically, if appropriate
Ascites	– Yes/No (if yes, small/medium/large volume subjectively assessed [10]))	– Drainage may be needed for large volume – Cytology may be warranted
Adnexal lesion	Description – Unilateral, bilateral, cystic, solid tissue, and other solid components, if evident. Statement of whether the mass demonstrates features of malignancy – Largest dimension – In adjacent organ infiltration is present	– Helpful for the surgical planning – Useful for surgical planning – Useful for surgical planning
Evidence of complications	– Bowel obstruction – Hydronephrosis – Venous obstruction/thrombosis – Pulmonary embolism	– May need emergency surgery, urologist consultation – May need to go for neo-adjuvant chemotherapy instead of primary surgery – It needs specific urgent treatment
Anatomical variants	– Abdominal vessels – Urinary tracts – Other visceral abnormalities	– Helpful for surgical planning to avoid damage
Others	– Exclude the presence of other malignancies that may be associated with peritoneal carcinomatosis, such as gastro-intestinal cancer or pancreatic cancer	– The treatment planning is different

No consensus was reached regarding the inclusion of the FIGO stage nor the use of surgical scores in the imaging report, such as the peritoneal cancer index [12], Fagotti score [13], or others. Furthermore, no consensus was reached about the use of RECIST criteria in the structured report, as these are mainly reserved for patients who are enrolled in clinical trials to monitor treatment response [14].

### Sites that may preclude optimal cytoreduction

The criteria for sites of disease that may preclude optimal cytoreduction may differ between centres, depending on the surgical expertise, patient’s performance status, morbidity of surgery, and intensive care facilities [15].

**Fig. 1:**
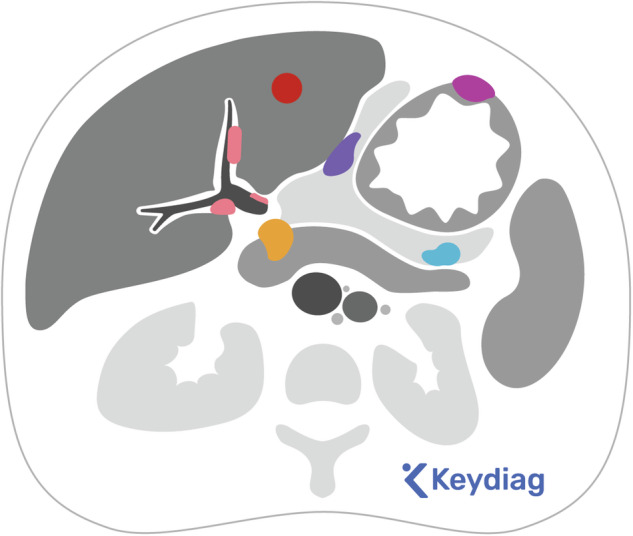
Sites of disease that may preclude optimal cytoreduction include: hepatic parenchyma metastases (red); metastases in the porta hepatis (pink); metastases in the lesser sac (blue) and lesser omentum (violet); metastases in the head of the pancreas (orange) and in the gastric wall (purple)

**Fig. 2:**
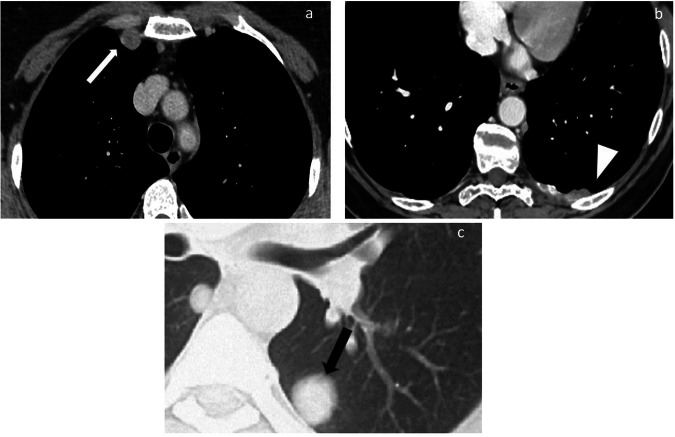
Thoracic sites of disease that may preclude optimal debulking include right internal mammary lymphadenopathy (white arrow in (**a**); pleural lesions (white arrowhead in (**b**)); lung metastases (black arrow in (**c**))

**Fig. 3:**
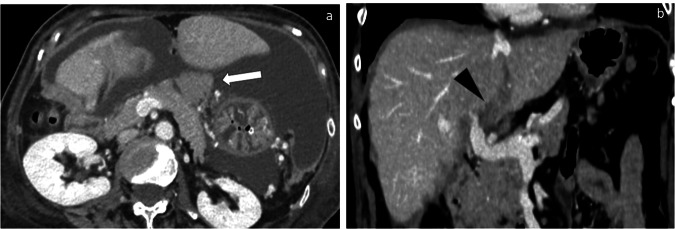
Axial (**a**) and coronal (**b**) CT images show infiltration of the lesser sac (white arrow) and of the hepatic hilum (black arrowhead)

**Fig. 4:**
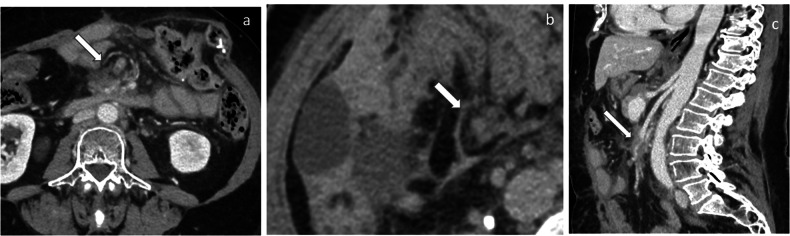
Small bowel mesentery Infiltration: axial (**a**, **b**) and sagittal (**c**) CT images of a diffuse deep infiltration (white arrows)

**Fig. 5:**
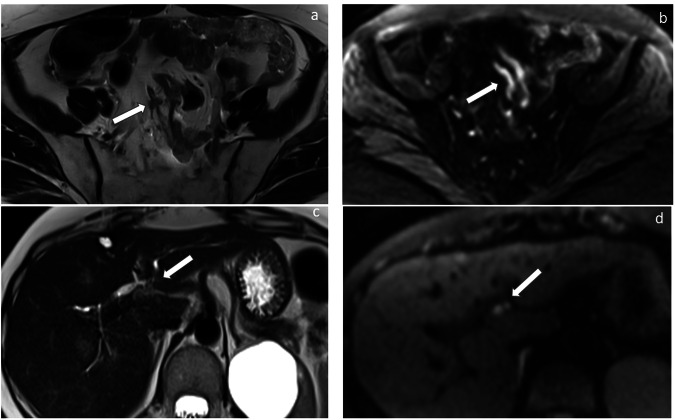
Abdominal MR images showing infiltration of the small bowel mesentery as hypointense thickening of the mesenteric surface on T2w images (**a**) and as an area with restricted diffusion on DWI MRI images (**b**); subtle infiltration of the hepatic hilum on axial T2-weighted (**c**) and high *b*-Value DWI MRI images (**d**). MRI can be used as a complement to CT as a problem-solving tool, like evaluating the extent of serosal and mesenteric involvement or bowel involvement

The radiological report should pay special attention to these sites to provide complete, clear, and relevant information for treatment decisions by the multidisciplinary team. The main sites that may preclude complete optimal cytoreduction [3, 15] and require specific mention in the report are summarised in Table 3, and some of them are represented in Figs. 1–5. In contrast to the 2010 published guidelines, the 2 cm diameter for difficult-to-resect sites is no longer considered valid [6]. Furthermore, criteria for patients who are not candidates for primary surgery [15] have been included.

**Table 3:** Sites of disease that may preclude optimal cytoreduction and require specific mention in the report

**Thoracic lesions**
Pulmonary nodules Thoracic lymphadenopathy (except for cardio-phrenic) Pleural metastases
**Abdominopelvic lesions**
Hepatic parenchyma (central or multisegmental) Pancreas (head or body) Lesser sac Mesentery (diffuse, multifocal, or mesenteric root involvement) Abdominal wall Bowel/stomach involvement (involving such large parts that resection would lead to a short bowel syndrome if the remaining bowel < 1.5 m) Upper abdominal lymphadenopathy (above the renal arteries) Inguinal lymph nodes Other visceral lesions

## Discussion

### Imaging techniques

#### Evaluation at diagnosis

The initial approach to investigating suspected adnexal masses is ultrasonography (US) which can accurately characterise approximately 80% of adnexal masses [16]. In cases where the US is inconclusive, MRI serves as the second-line technique for lesion characterisation, with O-RADS evaluation being recommended [8, 17].

When malignancy is suspected, the use of contrast-enhanced CT is advised for the evaluation of disease extent. CT is the imaging modality of choice because of its widespread availability, rapid imaging time, cost-effectiveness, and utility in the follow-up, providing consistent and reproducible results [18]. The portal venous phase is the recommended contrast phase for this purpose. To ensure high accuracy, CT should be performed using a multislice CT scanner, and multiplanar reconstructions should be reviewed to increase confidence in interpreting the scans [19].

A significant update to the guidelines is the recommendation to include chest CT for complete staging. It provides crucial information about potential extra-abdominal disease, impacting treatment planning. Notably, the CT protocol does not require specific preparation, such as fasting, oral contrast administration, or spasmolytic drugs [20]. Indeed, none of these preparations reached consensus.

The present updated ESUR guidelines recommend a CT scan as the initial evaluation for patients with advanced OC before debulking surgery, with the option to choose a combination of chest CT and MRI abdomen and pelvis as a valid alternative when iodine contrast media cannot be administered and in pregnant women. A specific question to the experts’ panel on pre-operative evaluation of young patients did not reach consensus about the imaging technique (i.e., not performing CT and only MRI) to use, therefore it can be adapted to the single centre’s preference and experience [21].

When used for pre-treatment evaluation, abdominal MRI should include T1- and T2-weighted imaging and diffusion-weighted imaging (DWI) of the abdomen and pelvis, with the administration of a spasmolytic drug, to minimise motion artefacts. Gadolinium-based contrast agents can be used to complete evaluation. The use of pineapple juice has been proposed as an adjunct to minimise the hyper T2 effect of the bowel lumen (Supplementary Table 1). MRI protocol may be adjusted based on the indication of the exam.

Whole-body (WB)-MRI may be considered for a site-based analysis of sub-centimetre lesions, where DWI sequences, combined with morphological sequences, may outperform CT [22] and [^18^F]FDG PET-CT [23] to detect challenging to resect sites, such as serosal and mesenteric involvement. However, this technique is less available, more expensive, longer, and thus harder for patients. Furthermore, it requires a lot more expertise to perform and interpret appropriately. Therefore, more studies should be performed to confirm WB-MRI superiority compared to the other techniques, before its implementation in the clinical routine, especially over abdominal and pelvic MRI.

[^18^F]FDG PET-CT has a limited role in the initial evaluation of patients with OC and is not recommended as a first-line imaging technique. Indeed, [^18^F]FDG PET-CT cannot differentiate reliably between borderline and benign tumours; it may fail to detect clear cell and mucinous invasive subtypes due to low FDG uptake as well as give false positive results in the presence of endometriosis and hydrosalpinxes [24, 25]. Although [^18^F]FDG PET-CT may be as accurate as CT in predicting the presence of chest metastasis, it fails to detect small peritoneal metastases [26], therefore its role in initial evaluation is limited to the evaluation or confirmation of possible extraperitoneal spread of disease. On the other hand, [^18^F]FDG PET-CT may be valuable in evaluating ambiguous extra-abdominal findings, such as extra-abdominal lymph nodes [27], that could influence treatment decisions.

#### Follow-up and recurrence

Post-treatment surveillance of OC typically involves a combination of clinical examination, routine CA-125 blood tests, and periodic imaging. There is currently no standardised clinical recommendation for imaging frequency, and radiological imaging may be indicated according to histology of the tumour, symptoms, clinical examination or rising of CA-125 level, or other markers [15]. The crucial issue is to perform the same imaging modality each time to be able to compare. Still, CT scans are commonly performed at intervals of three to six months or when recurrence is suspected based on clinical or laboratory findings [15].

Indeed our survey indicated that CT remains the primary imaging modality for follow-up and assessment of treatment response. The CT protocol for follow-up is the same as for evaluation at diagnosis.

To date, not enough studies have evaluated the role of MRI in terms of follow up and recurrence, therefore these guidelines cannot give any recommendation about its use during follow-up. However, the use of MRI for follow-up could be discussed in multidisciplinary teams for young patients to save radiation exposure.

[^18^F]FDG PET-CT may be beneficial in cases where CA-125 levels are elevated, but CT is negative. [^18^F]FDG PET-CT may also help detect persistent disease and notice distant sites when planning a secondary cytoreductive surgery [28]. Indeed, results of a retrospective multicentric trial demonstrated improved progression-free survival and a longer time to first subsequent therapy in OC patients that, at first recurrence, underwent secondary cytoreductive surgery, with no residual disease [29].

### Structured report

The use of a structured report for the pre-treatment evaluation of OC using CT or MRI is recommended [10, 30, 31], since it enhances uniformity in disease reporting and improves communication between radiologists and clinicians, potentially leading to better treatment choice and surgical planning and hence optimal patient care. This is particularly relevant as not all lesions are clearly visible at laparoscopy (behind the upper part of the liver, for example) and need to be removed if a primary surgery is decided.

A structured radiological report should provide comprehensive information about the primary tumour and its dissemination in the peritoneal cavity, the presence of lymph node enlargement, parenchymal involvement of thoracic, abdominal, and pelvic organs, and extra-abdominal tumour spreading. A study including 205 reports showed that the implementation of a synoptic report at the initial CT evaluation of patients with advanced OC, improved the overall documentation rate of disease extent from 39% for simple structured reports to 99% for synoptic reports and unresectable or challenging-to-resect sites from 37% to 100%, respectively [32].

The expert panel advises an organ-based disease description with detailed mapping of the peritoneal sites in the radiological report. The recommended structured report essential items are summarised in Table 2.

Compared to the 2010 guidelines [6], a description of vascular variants (such as the position of the left renal vein compared to the aorta) is now recommended to help surgeons avoid complications during lymph node dissection.

The definition of lymph nodes as metastatic has reached consensus, indicating positive lymph nodes in general with a short axis ≥ 10 mm, except for cardio-phrenic, retrocrural, and internal mammary lymph nodes, considered positive with a short axis ≥ 5 mm, and of inguinal lymph nodes considered positive with a short axis ≥ 15 mm [11]. Although different short axis sizes have been described in multiple papers [10, 33], in these guidelines, intended also for general practice radiologists, the expert panel decided to keep it simple and to adopt the node-RADS size criteria, that align with the 2010 guidelines [6, 11]. However, a discussion in the multidisciplinary meeting may be helpful about the significance of slightly enlarged extra-abdominal lymph nodes.

The inclusion of the FIGO/TNM classification, peritoneal cancer index, or other scores did not reach > 80% agreement. The gynaecological surgeons make the decision on how far a cytoreductive surgery can go, considering the patient’s performance status and disease-related factors, together with imaging evaluation of the disease extension. For this reason, the imaging criteria for sites that may preclude optimal cytoreduction in OC should always be discussed and approved in a multidisciplinary meeting [34]. There are also some locations indicating that patients may not be candidates for primary surgery, and these should be described in the report and possibly discussed during a multidisciplinary meeting.

Compared to the 2010 guidelines, the cut-off of 2 cm for the peritoneal supra-mesocolic and small bowel peritoneal metastases did not reach consensus and have been removed.

### Potential implementations in the future

Although not included in the recommendations of these guidelines, aimed at a practical approach, technological and computational developments that are underway are here mentioned, as they may have already a role in few centres or may be more spread out in the future.

#### PET-MRI

Combined PET-MRI has been available for clinical use since 2010; however, the number of installed PET-MRI systems is still small compared to clinical [^18^F]FDG PET-CT systems [24]. Today, PET-MRI is primarily performed for oncological indications. Still, its adoption has been challenged by the lack of protocol, workflow standardisation [24], and reimbursement, which is often non-existent or variable between countries [35]. Given the challenges of standardisation and harmonisation of MRI data acquisition, a clinical trial comparing this imaging modality to the existing ones is considered difficult to achieve. For this reason, a PET-MRI registry for pooling data acquired at multiple centres was created. In the specific clinical setting of OC, [^18^F]FDG PET-CT, and PET-MRI demonstrated accuracy rates of 71% and 92.5% in the peritoneal staging and characterisation of suspected OC [36]. In a group of 34 patients, PET-MRI was more accurate than DW-MRI (*p*  =  0.001) when evaluating patients at primary diagnosis, although no difference was noted in patients treated with chemotherapy. In the small bowel regions, there was a tendency towards higher sensitivity but lower specificity of PET-MRI compared to DW-MRI. Few published studies suggest the high diagnostic potential of PET-MRI for the assessment of the recurrence of female pelvic malignancies and higher diagnostic confidence in discrimination between benign and malignant lesions compared to [^18^F]FDG PET-CT [37]. In the future, further studies are suggested, possibly randomised and prospective, to assess the added value of the PET-MRI imaging modality.

#### Artificial intelligence/radiomics

Artificial intelligence (AI), a branch of computer science, refers to the ability of computer systems to learn from input data. AI is playing an essential role in many different areas of imaging. In recent years, AI-based multi-omics research has been widely conducted with a focus on OC [38]. Radiomics is part of the AI non-invasive approaches that aim to create prediction models by extracting quantitative features from medical images through specific steps, such as image acquisition, segmentation, feature extraction, and model construction [39]. Although many issues need to be addressed, primarily related to the quality of the studies and the possibility of routine clinical applications of predictive radiomic signatures [40], radiomics raises particular hope in OC to capture the whole disease heterogeneity better and offer a new tool to predict tumour aggressiveness, response to therapy and survival [41–45]. Furthermore, studies addressing the advances in research on molecular targeted therapies and how they may be reflected by imaging would be of great help in the future. The future of radiomics clinical application may rely on large multicentric studies, always including appropriate validation cohorts to ensure the reproducibility of models and performed according to high-quality standards [46].

In conclusion, the ESUR Female Imaging working group has updated the guidelines for imaging of patients with OC, recommending contrast-enhanced chest, abdominal, and pelvic CT for pre-treatment evaluation and follow-up. A structured report, including all sites of disease reported according to the SAR-ESUR lexicon, has been introduced; the sites of disease that may preclude an optimal cytoreduction are highlighted to pay special attention to them when reporting the CT exam and presenting the results to the multidisciplinary meetings, where the decision on primary debulking or interval debulking surgery should be undertaken.

## Supplementary information

ELECTRONIC SUPPLEMENTARY MATERIAL

## Abbreviations

ESUR: European Society of Urogenital Radiology
FIGO: International Federation of Gynaecology and Obstetrics
OC: Ovarian cancer
O-RADS: Ovarian-Adnexal Reporting and Data System
SAR: Society of American Radiology
TRUST: Trial of Radical Upfront Surgical Therapy in advanced ovarian cancer
US: Ultrasonography
WB: Whole-body
