# Supplementary material for: Ovarian cancer staging and follow-up: updated guidelines from the European Society of Urogenital Radiology female pelvic imaging working group
Source: Eur Radiol. 2025 Jan 11;35(7):4029–39. doi: 10.1007/s00330-024-11300-7 (PMC12165971; doi:10.1007/s00330-024-11300-7)
Supplement: Supplementary file 1 — ELECTRONIC SUPPLEMENTARY MATERIAL [file 330_2024_11300_MOESM1_ESM.pdf]

Ovarian cancer staging and follow-up: updated guidelines from the European Society of Urogenital Radiology Female Pelvic Imaging working group

ELECTRONIC SUPPLEMENTARY MATERIAL

Supplementary Table 1. Details on CT and MRI protocols

CT	
Hardware requirement	Multislice CT (>16 slices)
Preparation	No fasting
	No spasmolytic agent
	No oral contrast
Coverage	Thorax*, abdomen and pelvis
Contrast phases	Portal-venous phase
	Non contrast, arterial and delayed phases did not reach consensus.
	If low-grade is suspected: non-contrast phase may be indicated to detect calcified peritoneal lesions
Post-processing	Coronal and sagittal reconstructions

MRI	
Hardware requirement	Field strength at least 1.5T or higher
Preparation	No fasting
	No voiding urinary bladder before the exam
	Do not schedule according to menstrual cycle
	No vaginal or rectal opacification
	Use of spasmolytic agent is recommended
Coverage	Abdomen and pelvis

Sequence	Plane	Specifications
T2 without fat saturation (FS) of the pelvis	Axial	Slice thickness ≤5 mm
T2 without FS of the pelvis	Sagittal	Slice thickness ≤4 mm
T1 without FS of the pelvis	Axial	Slice thickness ≤5 mm
T1 with FS of the pelvis	Axial	Slice thickness ≤5 mm
DWI of the pelvis	Axial	Slice thickness ≤5 mm

		B-value of 0-50 and 800-1000
T2 without FS of the abdomen	Axial	Slice thickness ≤5 mm
T1 without FS of the abdomen	Axial	Slice thickness ≤5 mm
DWI of the abdomen	Axial	Slice thickness ≤5 mm<br>B-value of 0-50 and 800-1000
Contrast enhanced T1 of the pelvis	Axial	Slice thickness ≤3 mm
Contrast enhanced T1 of the abdomen	Axial	Slice thickness ≤3 mm

Supplementary Table 2. Structured report highlighting the changes compared to the 2010 guidelines [6]

**2010 structured reporting [6]**	**2024 reporting aligned to [10]**
Adnexa	
Size, morphology and uni- or bilaterality of the ovarian mass with a statement of whether the mass demonstrates features of malignancy	Description of adnexal lesion: unilateral, bilateral, cystic, solid component (solid tissue; other solid components). Statement of whether the mass demonstrates features of malignancy
Uterine endometrial thickening	//
Peritoneal carcinomatosis	
Bladder, bowel invasion or pelvic side-wall invasion	Peritoneal carcinomatosis in the pelvis: paravesical spaces, bowel, pelvic sidewall, pouch of Douglas
Omental metastases	Greater Omentum
Site and size of other peritoneal/serosal implants outside the pelvis	Peritoneal carcinomatosis outside the pelvis, including the paracolic gutters
Involvement of the small bowel mesentery	Mesentery (Root of mesentery involvement; pattern: nodular or infiltrative (retractile)
Supracolic sites of disease including the gastrohepatic, gastrosplenic and splenocolic ligaments	Gastrocolic ligament<br>Gastrosplenic ligament<br>Lesser omentum<br>Lesser sac
Surface, subcapsular or parenchymal metastases	Subdiaphragmatic lesions<br>Perihepatic lesions

	Perisplenic lesions
Metastatic disease other than peritoneal carcinomatosis	
Invasion of the abdominal wall	Abdominal wall
Liver and spleen metastases	Hepatic parenchyma<br>Splenic parenchyma
	Pulmonary nodules<br>Pleuric nodules<br>Other visceral lesions
Lymph nodes	
Cardiophrenic lymph nodes with a short-axis diameter of >5 mm<br><br>Site of lymph nodes with a short-axis diameter of >1 cm, or suspicious clusters of smaller lymph nodes	*Thoracic lymphadenopaties (short axis ≥10 mm)*<br>Supraclavicular, mediastinal, hilar, axillary (short-axis diameter ≥10 mm)<br>Internal mammary, cardio-phrenic, retrocrural lymph nodes (short axis ≥5 mm<br><br>*Abdominal lymph nodes (short axis >10 mm)*<br>Abdominal lymph nodes (short axis ≥10 mm)<br>- Upper abdominal (above the renal arteries)<br>- Retroperitoneal (infra-renal para-aortic, intercavoaortic)<br>- Mesenteric<br>- Pelvic<br><br>Inguinal lymph nodes (short axis ≥15 mm)
Fluids collection	
Presence and size of pleural effusion	Pleural effusion volume (small/large or small/medium/large)
Ascites in the pelvis or upper abdomen, and amount of ascites	Ascites volume (small/large or small/medium/large)
Complications	
Evidence of complications such as bowel obstruction, hydronephrosis or venous obstruction/thrombosis	Evidence of complications such as bowel obstruction, hydronephrosis or venous obstruction/thrombosis
